# A Hybrid Bionic Image Sensor Achieving FOV Extension and Foveated Imaging

**DOI:** 10.3390/s18041042

**Published:** 2018-03-30

**Authors:** Qun Hao, Zihan Wang, Jie Cao, Fanghua Zhang

**Affiliations:** 1 School of optics and photonics, Beijing Institute of Technology, Key Laboratory of Biomimetic Robots and Systems, Ministry of Education, Beijing 100081, China; qhao@bit.edu.cn (Q.H.); 3120120280@bit.edu.cn (Z.W.); 3120160316@bit.edu.cn (F.Z.); 2 NUS Suzhou Research Institute (NUSRI), Suzhou Industrial Park, Suzhou 215123, China

**Keywords:** artificial compound eyes, foveated imaging, super-resolution, Risley prisms

## Abstract

Based on bionic compound eye and human foveated imaging mechanisms, a hybrid bionic image sensor (HBIS) is proposed in this paper to extend the field of view (FOV) with high resolution. First, the hybrid bionic imaging model was developed and the structure parameters of the HBIS were deduced. Second, the properties of the HBIS were simulated, including FOV extension, super-resolution imaging, foveal ratio and so on. Third, a prototype of the HBIS was developed to validate the theory. Imaging experiments were carried out, and the results are in accordance with the simulations, proving the potential of the HBIS for large FOV and high-resolution imaging with low cost.

## 1. Introduction

Compound eyes and human eyes have been studied in regard to their remarkable properties for optical imaging [1,2,3,4,5]. Compound eye provides a large field of view (FOV), infinite depth of field (DOF), low aberrations and motion acuity, which can be used in many applications, such as high speed motion detection, large FOV surveillance and machine vision [6,7,8,9]. Artificial compound eyes can be divided into two types, namely planar artificial compound eyes (PACE) and curved artificial compound eyes (CACE). PACE [10,11,12] are mainly designed for super-resolution imaging, which use the sub-pixel shifts among the ommatidia to resample the scene with a relatively high spatial sampling frequency. For a typical PACE, the optical axes of ommatidia are parallel, and the FOVs of ommatidia are identical, so it is difficult to obtain large whole FOV with PACE. CACE [1,3,13] are mainly designed for FOV extension. Compared with PACE, the overlaps between FOVs of adjacent ommatidia of CACE are usually small enough to extend to the whole FOV while avoiding a blind zone. Therefore, the image resolution of CACE is lower than PACE under the same parameters of individual ommatidium. In order to obtain both large FOV and high resolution, the Aware-2 imaging system [14] utilizes a multi-scale lens and a micro-camera array achieving 120° and 38 μrad instantaneous FOV of a single pixel, but the system is bulky, and it is time-consuming due to the required calibration of 98 micro-cameras and the sub-image mosaic with iterative methods. In addition, large volume redundant data results in low efficiency for object detection or target tracking [15]. In recent years, the development of a flexible printed circuit board and liquid lens has motivated some remarkable CACE designs [1,16,17,18]. Each ommatidium on those sensors has one pixel in common, so they can achieve a large FOV while keeping relatively low spatial resolution. To improve the image quality, Woong-Bi Lee et al. [13] Utilized a digital signal processing algorithm to reconstruct images with high resolution, but the images including 16 × 16 pixels could not meet requirements. Also, some scanning methods have been proposed to enlarge the FOV of a single aperture or improve the image quality of CACE, but the imaging procedure is time-consuming [1,19,20], and the resolution of the image is still limited by the small volume of pixels.

Foveated vision is inspired by human eyes, and it provides a practical solution to achieve a large FOV (peripheral imaging) and high resolution (fovea imaging) [4,21,22,23,24] with low data redundancy. The foveal ratio is defined as the ratio between the spatial sampling frequencies of the fovea and the periphery. A large foveal ratio of foveated vision is attractive for many applications, such as medical facilities, surveillance, and robot navigation [4,21,25]. However, compared with CACE, the whole FOV is determined by peripheral imaging of a single image sensor, and the peripheral view also needs acceptable resolution for automatic visual tasks. Therefore, the tradeoff between FOV and the resolution of foveated vision needs to be further resolved.

Recently, some researchers have been studying the combination of a compound eye and foveated vision to improve the performance of imaging systems. Guillem Carles et al. [15] developed a multichannel imaging system which combines prism array and PACE to obtain FOV extension and foveated imaging. However, in order to achieve a high foveal ratio in the fovea, the FOV of each ommatidium overlaps in the fovea region, and the system only achieves two-fold FOV extension with a 5 × 5 camera array. Kang Wei et al. [26] designed a reconfigurable polymeric optofluidic device with an array of integrated millimeter-sized fluidic lenses, which combines the large FOV of CACE and the adaptive focusing capabilities of the human eye to achieve FOV and DOF extension. Xiongxiong Wu et al. [27] simulated a micro-camera array to achieve large FOV imaging like in a compound eye, with a foveated imager located behind each micro-camera to obtain high resolution in the regions of interest (ROI). However, compared with PACE, the spatial resolution is not improved. A combined method was proposed in our previous work [28], which possesses the large FOV characteristic of CACE and the retina-like feature of human vision. To the best of our knowledge, there is currently no practical imaging method that can obtain FOV extension and super-resolution simultaneously.

To solve the problems above, a hybrid bionic image sensor (HBIS) is proposed in this paper. Features of PACE, CACE and foveated vision are integrated to achieve FOV extension (by CACE), super-resolution (by PACE) of ROI and a large foveal ratio (foveated vision). The FOV is extended efficiently with a CACE structure. Foveated imaging by CACE and PACE allows a higher resolving power and larger FOV than traditional foveated vision. Risley prisms are widely used for accurate and fast beam scanning and pointing [29,30], and these are employed in front of the central ommatidium to imitate the movement of the fovea and generate sub-pixel shifts of sub-images for super-resolution reconstruction. A prototype is developed to test the features of HBIS by carrying out experiments with outdoor and indoor scenes. The experimental results show consistency with the theoretical analysis and its potential for a large FOV and foveal ratio at a low cost.

## 2. Methods

**Figure 1:**
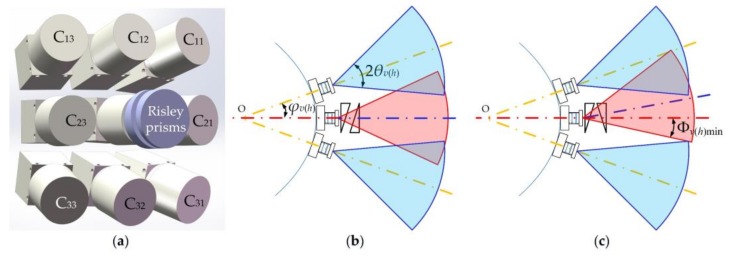
Schematic design of the hybrid bionic image sensor (HBIS); (**a**) the basic structure of the HBIS; (**b**) the situation in which the thin end of one prism is aligned with the thick end of the other prism; (**c**) the situation in which the two prisms are aligned, and the thick ends are oriented to vertical or horizontal directions.

The proposed HBIS integrates features of PACE, CACE and foveated vision. Here, we only utilized a 3 × 3 ommatidia array to illustrate the method; a HBIS with more ommatidia and larger FOV also could be developed based on the proposed method. The schematic diagram of HBIS is shown in Figure 1, where C*_ij_* is the sequence of each camera, and *i* and *j* denote the row and column numbers respectively. The red and yellow dot-dashed lines are the original optical axes of the cameras, and the blue dot-dashed line is the optical axis of the central ommatidium that is deviated by the Risley prisms, Φ*_v_*_min_ and Φ*_h_*_min_ denote the minimum angles between the edge of the FOV of central ommatidium (the edge closer to the red dot-dashed line) and the red-dashed line in the vertical and horizontal directions with the two prisms aligned, *φ_v_* and *φ_h_* denote inclined angles between the original optical axis of the central ommatidium and the optical axes of the peripheral ommatidia of C_12_/C_32_ and C_21_/C_23_, respectively. *θ_v_* and *θ_h_* denote half FOVs in the vertical and horizontal directions of the individual ommatidium. Risley prisms are composed of two identical prisms which can be rotated independently. 

Firstly, HBIS is composed of a camera array distributed on a curved surface like CACE in an apposition compound eyes pattern [31]. Secondly, Risley prisms are controlled to be fast and accurate, to shift the images of the central ommatidium, that is, the fovea, in sub-pixel accuracy for super-resolution imaging of PACE. Thirdly, super-resolution imaging allows the fovea to have a higher spatial sampling frequency than the periphery, which achieves foveal imaging.

It should be noted that only the case with the original optical axis of the central ommatidium involved in the deviated FOV of the central ommatidium is studied here, and other cases are discussed in the discussion section.

### 2.1. FOV Extension

The HBIS works in two stages. In stage one, all of the ommatidia detect objects in their FOV independently, and the FOV of the fovea is fixed without scanning by Risley prisms. Once an object is detected by any ommatidia, the HBIS goes into stage two. In stage two, the FOV of the fovea is coarsely adjusted by rotating Risley prisms to stare at the object immediately. Then, the Risley prisms are precisely rotated over a small range to achieve imaging with sub-pixel shifts for super-resolution reconstruction. In the meantime, all the periphery ommatidia keep detecting objects in their own FOV to avoid missing objects. The HBIS will go back to stage one when the object moves out of the scan field of the fovea.

The same optical system and image sensor chip are employed for all of the ommatidia to reduce the cost and design complexity. The design of a CACE structure should ensure partial overlaps between adjacent ommatidia to avoid a blind zone wherever the fovea is “watching”. In Figure 1b, the phases of the two Risley prisms are inverse, which causes the optical axis of the fovea to be unchanged. Therefore, the condition of partial overlap is expressed as

(1) $$\phi_{v\left(h\right)}\leq 2\theta_{v\left(h\right)}.$$

In Figure 1c, the phases of the two Risley prisms are identical, and the deviation angles of the optical axis of the fovea achieve maximum values in the vertical and horizontal directions. To avoid a blind zone, the structure should satisfy the condition

(2) $$\Phi_{v\left(h\right)\min}+\theta_{v\left(h\right)}\geq \phi_{v\left(h\right)}.$$

It is obvious that Equation (2) is tougher than Equation (1) because the fixation points of the periphery ommatidia are fixed. When the fovea steers its FOV away from the center of the whole FOV of HBIS, as in Figure 1c, the FOVs of the periphery ommatidia need to be closer to the center of the whole FOV of HBIS than they need to be in Figure 1b. It can also be observed that the large scan field of the fovea makes the FOVs of the periphery ommatidia close to the center of HBIS, resulting in a small whole FOV of the HBIS. Hence, the value of *φ_v_*_(*h*)_ should be configured according to the values of Φ*_v_*_min_ and Φ*_h_*_min_.

**Figure 2:**
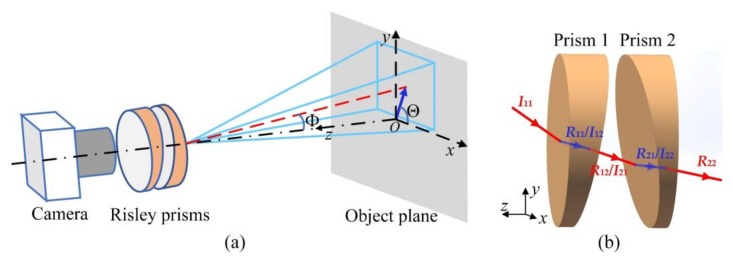
The central ommatidium imaging (**a**) and ray tracing (**b**) models with Risley prisms. Risley prisms are located close to the entrance pupil of the optical system of the central ommaditium. The red lines of (**b**) represent light beams in the air, and the blue lines are light beams inside the prisms.

The imaging model with Risley prisms is illustrated in Figure 2a, where the dot-dashed line is the optical axis of the camera, the red dashed line is the deviated optical axis by the Risley prisms, Φ is the deviation angle of the optical axis, and Θ is the azimuth angle of the optical axis. To find the maximum values of Φ in the vertical and horizontal directions, the two prisms are aligned, and the phase angles are adjusted to 90° and 180°. Then non-paraxial ray tracing is utilized to calculate the deviation angles as follows.

Refractions occur on four surfaces of the Risley prisms. Figure 2b shows the case of refractions with the two prisms aligned, where ***I*** is the vector of incident light from one pixel, and ***R*** is the vector of emergent light. The two subscripts of ***I*** and ***R*** indicate the sequence of prisms and the sequence of surfaces of each prism, respectively.

Given a pixel (*X*, *Y*) located in the pixel array with *M* × *N* pixels, ***I***_11_ is calculated as

(3) $$I_{11}=\left(\frac{p\left(Y-n_{0}\right)}{\sqrt{p^{2}\left[{\left(Y-n_{0}\right)}^{2}+{\left(X-m_{0}\right)}^{2}\right]+f\prime^{2}}};\frac{p\left(X-m_{0}\right)}{\sqrt{p^{2}\left[{\left(Y-n_{0}\right)}^{2}+{\left(X-m_{0}\right)}^{2}\right]+f\prime^{2}}};\frac{f\prime }{\sqrt{p^{2}\left[{\left(Y-n_{0}\right)}^{2}+{\left(X-m_{0}\right)}^{2}\right]+f\prime^{2}}}\right),$$

where *p* is the pixel pitch, f′ is the focal length of the optical system, and (*m*_0_, *n*_0_) represents the center of the pixel array. The normal vectors of the four surfaces are calculated as:

(4) $$\left\{ \begin{array}{l} n_{11}=(0;0;1)\\ n_{12}=(-\sin\alpha \cos\phi_{1};-\sin\alpha \sin\phi_{1};\cos\alpha )\\ n_{21}=(\sin\alpha \cos\phi_{2};\sin\alpha \sin\phi_{2};\cos\alpha )\\ n_{22}=(0;0;1) \end{array}\right.,$$

where *α* is the wedge angle of the Risley prisms, $\phi_{1}$ and $\phi_{2}$ are phase angles of prism 1 and prism 2—that is, the angles from the *x* axis to the thin ends of the prisms in an anticlockwise direction. Following Snell’s law in vector form [29], the emergent light, *R*_22_, through the Risley prisms is obtained. Then, the deviation angle, Φ, and the azimuth angle, Θ, as illustrated in Figure 2a, can be calculated with *R*_22_.

Substituting *X* = 1:*M*/2, *Y* = 1 and $\phi_{1}=\phi_{2}=180{}^{\circ}$ into the non-paraxial ray tracing method above, the minimum inclined angle between the right edge of FOV and the negative direction of the *z*-axis is obtained as Φ*_h_*_min_. In the same way, the minimum inclined angle Φ*_v_*_min_ between the lower edge of FOV and the negative direction of the *z*-axis can be obtained with *X* = 1, *Y* = 1:*N*/2 and $\phi_{1}=\phi_{2}=270{}^{\circ}$. Then, the conditions of Equation (2) can be fulfilled using the values of Φ*_v_*_min_ and Φ*_h_*_min_.

The inclined angles between the optical axis of the fovea and the optical axes of the corner ommatidia are deduced with $\varphi_{c}=\tan^{-1}\sqrt{\tan^{2}\varphi_{v}+\tan^{2}\varphi_{h}}$.

The whole FOV of the HBIS in the vertical and horizontal directions is *FOV_v_*_(*h*)_ = 2(*φ_v_*_(*h*)_ + *θ_v_*_(*h*)_), and the FOV is extended by 2*φ_v_*_(*h*)_.

### 2.2. Super-Resolution

**Figure 3:**
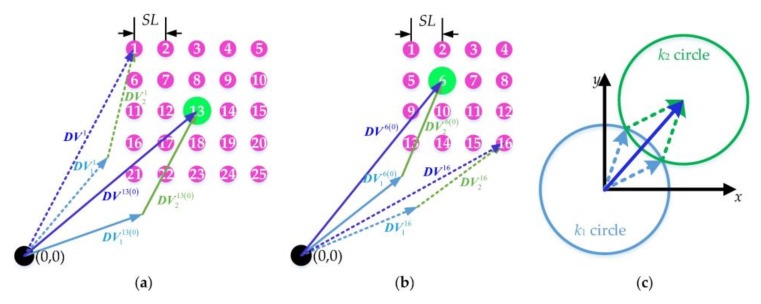
Sub-pixel scan patterns of (**a**) *h* = 5 and (**b**) *h* = 4, and (**c**) the model for inverse solutions of Risley prisms.

To obtain super-resolution imaging of PACE, multiple images with sub-pixel shifts are sampled by the fovea. It has been proven that the pixels always shift along with the optical axis [29]. In addition, sub-pixel shifts of the images need only fine adjustment of Risley prisms over a small range. Therefore, we assume that the Risley prisms produce identical shifts for the optical axis and pixel array. For practical use, we only need to shift the optical axis of the fovea in sub-pixel accuracy. For a commercial, off-the-shelf camera whose resolution is limited by pixel size, super-resolution techniques can improve the spatial resolution to approximate the diffraction limit. The optical spatial cutoff frequency of a diffracted limitation is derived as *v_o_* = *D*/1.22*𝜆f’*, where *D* is the entrance pupil [32] diameter, and *λ* is the optical wavelength. The Nyquist frequency of the pixel array is *v_p_* = 1/2*p*. Assuming *H* = *v_o_*/*v_p_*, the spatial resolution of the camera can be improved up to *H* times with super-resolution techniques, theoretically. *H* is the ratio of *v_o_* and *v_p_*, and it is a constant for a given imaging system with a fixed focal length. The parameter, *h* (*h* ≤ *H*), is viewed as a resolution improvement factor. The step length of the sub-pixel shift in the image plane is *sl* = *p*/*h*, where *h* is an integer. Given the object’s distance, *v*, the step length of sub-pixel shifts in the object’s plane is *SL* = *sl*∙*v*/*f’*. Figure 3a,b shows the scan pattern of the optical axis with odd and even values of *h*, respectively. The numbers on the circle dots denote the sequences of sub-pixel points. The green circle dots represent the intersection of the object’s plane and the optical axis with the initial phase angle. The purple circle dots are arranged by referring to the green dots.

The deviation vectors of the two prisms, $DV_{1}^{i}$ and $DV_{2}^{i}$, are deduced with

(5) $$\left\{ \begin{array}{l} DV_{1}^{i}=(-k_{1}\cos\phi_{1}^{i};-k_{1}\sin\phi_{1}^{i})\\ DV_{2}^{i}=(-k_{2}\cos\phi_{2}^{i};-k_{2}\sin\phi_{2}^{i}) \end{array}\right.,$$

where $k_{1}$ and $k_{2}$ are norms of the two prisms, and the superscript, *I*, refers to the index of the sub-pixel point in Figure 3a. The total deviation vector by the Risley prisms is

(6) $$DV^{i}=DV_{1}^{i}+DV_{2}^{i}=(-k_{1}\cos\phi_{1}^{i}-k_{2}\cos\phi_{2}^{i};-k_{1}\sin\phi_{1}^{i}-k_{2}\sin\phi_{2}^{i}).$$

$DV^{i}$ can also be deduced as

(7) $$DV^{i}=\left\{ \begin{array}{l} DV^{0}+\frac{sl\cdot v}{f\prime }\left(\mathrm{mod}\left(i,h\right)-\frac{h+1}{2};\mathrm{floor}\left(i/h\right)-\frac{h+1}{2}\right),\;\mathrm{when}\;h\;\mathrm{is}\;\mathrm{odd};\\ DV^{0}+\frac{sl\cdot v}{f\prime }\left(\mathrm{mod}\left(i,h\right)-\frac{h}{2};\mathrm{floor}\left(i/h\right)-\frac{h}{2}\right),\;\mathrm{when}\;h\;\mathrm{is}\;\mathrm{even}. \end{array}\right.,$$

where ***DV***^0^ is the deviation vector of the initial optical axis before the sub-pixel shift. In the example shown in Figure 3a, ***DV***^0^ = ***DV***^13^.

Substituting ***I***_11_ = (0,0,−1) into the non-paraxial ray tracing method, the deviation angle, Φ, and the azimuth angle, Θ, of the optical axis can be obtained, and ***DV***^0^ can be computed as (*v*∙tan Φ∙cos Θ; *v*∙tan Φ∙sin Θ). Then, *k*_1_ and *k*_2_ can be calculated as

(8) $$\left\{ \begin{array}{l} \left[\begin{array}{l} k_{1}\\ k_{2} \end{array}\right]={\left[\begin{array}{l} -\cos\phi_{1}^{0}-\cos\phi_{2}^{0}\\ -\sin\phi_{1}^{0}-\sin\phi_{2}^{0} \end{array}\right]}^{-1}\cdot DV^{0},\;\mathrm{when}\;\phi_{1}^{0}\neq \phi_{2}^{0}\\ k_{1}=k_{2}=\frac{1}{2}\left|DV^{0}\right|,\;\mathrm{when}\;\phi_{1}^{0}=\phi_{2}^{0} \end{array}\right..$$

According to the model illustrated in Figure 3c, and given the ***DV****^i^* and norms (*k*_1_ and *k*_2_) of $DV_{1}^{i}$ and $DV_{2}^{i}$, the two phase angles $\phi_{1}^{i}$ and $\phi_{2}^{i}$ can be computed as

(9) $$\left\{ \begin{array}{l} \phi_{1}^{i}=\phi_{0}^{i}\pm \arccos\left(\frac{k_{0}^{2}+k_{1}^{2}-k_{2}^{2}}{2k_{0}k_{1}}\right)\\ \phi_{2}^{i}=\phi_{0}^{i}\mp \arccos\left(\frac{k_{0}^{2}+k_{2}^{2}-k_{1}^{2}}{2k_{0}k_{2}}\right) \end{array}\right..$$

Two sets of the inverse solutions are obtained, and the one closer to the initial phase angles, $\phi_{1}^{0}$ and $\phi_{2}^{0}$, is adopted.

Once the optical axis scans over one sub-pixel point, an image is sampled. After all the sub-pixel points have been scanned, a complete set of sub-images with sub-pixel shifts is obtained. Then, a feature-based image registration [12] and an interpolation method of scatter pixel points [15] are utilized for super-resolution reconstruction.

### 2.3. Foveated Imaging

Super-resolution imaging is only realized by the fovea, where spatial-variant resolution is formed over the entire FOV of HBIS, which resembles foveated vision. What is more, the foveal ratio of the proposed HBIS is adjustable over a large range, which is determined by the maximum resolution improvement factor, *H*. This means the resolution of the fovea is modulated by the needs for specific tasks, such as object detection, recognition or target tracking, etc.

In addition, the ROI imaged by the fovea can be redirected by adjusting the optical axis using Risley prisms. This ability is like the fovea movement of human eyes. In contrast to the accurate inverse solutions for super-resolution, ROI only needs an approximate inverse solution. Therefore, the paraxial model [33] is enough. The scan range of the fovea is defined by rotating the Risley prisms from 0° to 360° with the two prisms aligned.

## 3. Simulations and Analysis

### 3.1. FOV Extension

**Figure 4:**
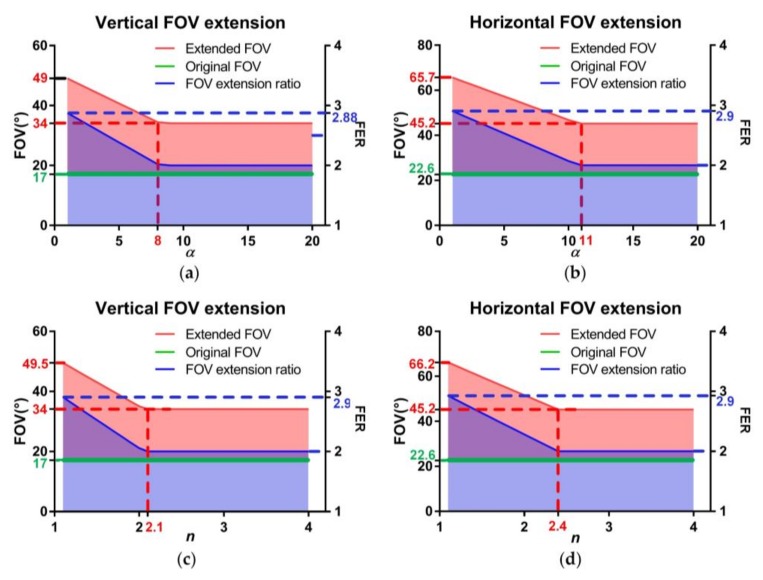
Vertical and horizontal whole field of view (FOV) and FOV extension ratio (FER) versus the wedge angle, *α*, and the refractive index, *n*. For (**a**,**b**), *n* = 1.5; for (**c**,**d**), *α* = 4°.

The FOV extension ratio (FER) is defined as the ratio between the whole FOV of the HBIS and the individual FOV of a single ommatidium. FER is viewed as the key indicator of HBIS, because we aim to demonstrate the capability of extending the FOV from one single aperture. Using given parameters of a pixel array and optical systems, the whole FOV can be determined by the parameters of Risley prisms, according to Section 2.1. We assume that the pixel pitch (*p*) is 3.75 μm, *M* × *N* is 960 × 1280, the focal length (*f*’) is 12 mm, the F-number (*F*) is 1.4, the object distance (*v*) is 50 mm and the resolution improvement factor (*h*) is 7. These parameters are unchanged for the rest of the simulations unless otherwise instructed. Based on the above methods, the results for the whole FOV and FER versus the refractive index, *n*, and the wedge angle, *α*, are shown in Figure 4. The red and blue shades represent the ranges of the whole FOV and FER. The upper limits of the shades correspond to Φ*_v_*_(*h*)min_ + *θ_v_*_(*h*)_ = *φ_v_*_(*h*)_ from Equation (2), and the lower limits correspond to *φ_v_*_(*h*)_ = *θ_v_*_(*h*)_.

In Figure 4, the trends of the curves (upper limits) are similar—that is, when *α* and *n* are small, the whole FOV and FER increase linearly with the growth of *α* and *n* until they reach the inflection points, and they are constants after the inflection points. When *α* is 1° (the smallest value employed), the optical axis of the fovea can only be slightly deviated, which means the scan field of the fovea is the smallest as well. So, the blind zone can be avoided without the FOVs of the periphery ommatidia designed getting too close to the center, leading to FER*_v_* = 2.88 and FER*_h_* = 2.9, maximumly. When *α* is 11°, the inflection point occurs, as shown in Figure 4b, which corresponds to the situation in which the right edge of FOV of the fovea is parallel to the initial optical axis before the Risley prisms when $\phi_{1}=\phi_{2}=180{}^{\circ}$. In this situation, the scan field of the fovea covers the whole FOV of the HBIS, and only the overlaps among the periphery ommatidia need to be ensured with *φ_h_* ≤ *θ_h_*. *φ_h_* = *θ_h_* corresponds to FER*_h_* = 2. Regarding *α* = 9° in Figure 4a, *n* = 2.2 in Figure 4c and *n* = 2.4 in Figure 4d, the inflection points occur in the same reason, and they also have a two-fold FOV extension in respective directions.

In Figure 4, it is noted that the values of *α* and *n* at inflection points in horizontal direction are larger than that in vertical direction. Because the condition for inflection points in horizontal direction is *φ_h_* = *θ_h_*, and *φ_v_* = *θ_v_* is the condition for inflection points in vertical direction. As *θ_h_* > *θ_v_*, the inflection points in horizontal direction are with larger *α* and *n* than the inflection points in vertical direction.

### 3.2. Imaging with Sub-Pixel Shifts for Super-Resolution

**Figure 5:**
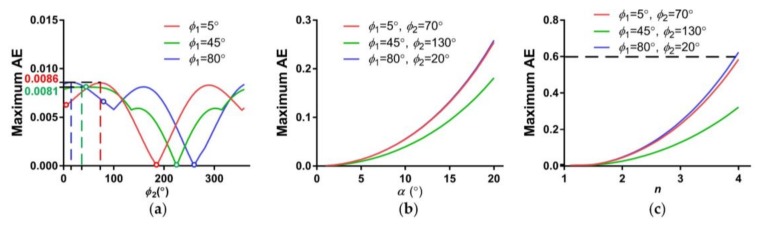
The maximum alignment error (AE) versus (**a**) initial phase angles, (**b**) wedge angle (*α*) and (**c**) refractive index (*n*).

To verify the proposed super-resolution imaging model, simulations of a sub-pixel scan of the optical axis are carried out. We assume that *α* is 4° and *n* is 1.5. The alignment error (AE) is defined as the deviation ratio between the simulated points and the ground truth, which is calculated as the ratio between the misalignment distance and the step length (*SL*) of the sub-pixel shifts. A large AE results in high redundancy of the multiple samplings in the scene. The maximum AE among the sub-pixel points is utilized to evaluate the data efficiency of the proposed method. The maximum AE varies with the initial phase angles, the refractive index (*n*) and the wedge angle (*α*), and the simulation results are shown in Figure 5.

In Figure 5a, simulations with $\phi_{1}=\phi_{2}$ and $\left|\phi_{1}-\phi_{2}\right|=180{}^{\circ}$ are avoided, points of which are marked by color circles in Figure 5a, because the cases with $\phi_{1}=\phi_{2}$ cannot achieve the sub-pixel patterns of Figure 3, and the cases with $\left|\phi_{1}-\phi_{2}\right|=180{}^{\circ}$ lead to control singularities which make it difficult to achieve a fast sub-pixel scan. From the curves, we can see that the maximum AE is below 0.01. The trends of the curves with $\phi_{1}=5{}^{\circ}$ and $\phi_{1}=45{}^{\circ}$ present approximate periodicity, because the scan pattern illustrates a square matrix, which is symmetrical about the directions 0°, 45° and 90°. Figure 5b,c present similar laws—that is, the maximum AE increases with the refractive index (*n*) and the wedge angle (*α*). The parameters are set as *α* = 4° in Figure 5b and *n* = 1.5 in Figure 5c. To sum up, the maximum AE always maintains relatively low values for the given parameters.

Taking the initial phase angles (5°,70°), (45°,130°) and (80°,20°) with *α* = 4° and *n* = 1.5 as examples, we further study performances of the HBIS for sub-pixel shifts. Based on the scanpath of Figure 3, the maximum ranges of phase angles for the three examples are 0.60°, 0.60° and 0.63°, respectively, which are very small and make fast sub-pixel scans possible. The maximum AEs of the pixel array of the three examples are 0.56, 0.53 and 0.49. The average AEs of the pixel array of the three examples are 0.071, 0.070 and 0.072, respectively. The maximum AEs of the optical axes of the three examples are 0.0085, 0.0061 and 0.0086, respectively. The maximum AEs of the pixel array are much larger than the average AEs of the pixel array, because a few pixels in the edge or corners of pixel array are deviated far more than other pixels closer to the center of pixel array. Even so, pixels with an AE of less than 0.6 only slightly decrease the capacity to super-resolve the scene [15].

In general, AEs of the whole pixels and the optical axis have relatively small values. In particular, the differences between the average AEs of the pixel array and the maximum AEs of the optical axis are no more than 0.07, verifying the consistency between the sub-pixel shifts of the optical axis and the pixel array and the effectiveness of the proposed super-resolution reconstruction method.

### 3.3. Foveated Imaging

Super-resolution imaging of the fovea and the original resolution imaging of the periphery ommatidia give rise to foveated imaging. The foveal ratio and the bandwidth saving ratio (BSR) are basic indicators of foveated vision. The resolution of the fovea is adjustable according to the needs of variable tasks and the specific circumstances. The foveal ratio is defined as the ratio between spatial sampling frequencies of the central and periphery ommatidia, which is equal to resolution improvement factor, *h*, of the fovea. BSR is calculated as

(10) $$\mathrm{BSR}=\left(1-\frac{1}{h^{2}}\right)\left(1-\frac{1}{{\mathrm{FER}}_{v}{\mathrm{FER}}_{h}}\right).$$

**Figure 6:**
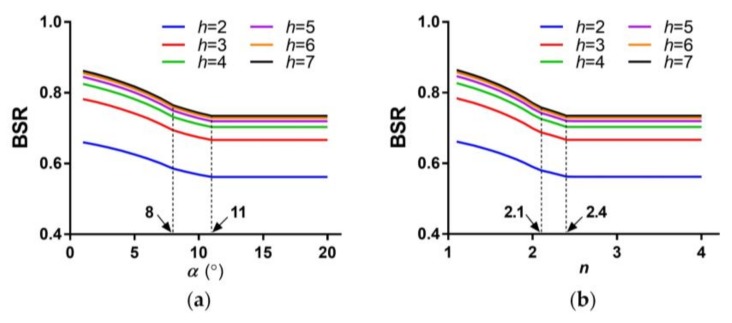
Bandwidth saving ratio (BSR) versus (**a**) the wedge angle, *α*, and (**b**) the refractive index, *n*.

From the definition of BSR, we can see that BSR is determined by *h* and FER. From the above analysis, we know that FER is mainly affected by *n* and *α*. Figure 6 shows the results of BSR versus the wedge angle, *α*, and the refractive index, *n*, respectively. The trends of the curves are similar to the curves of Figure 4, and the two inflection points correspond to the inflection points in Figure 4. BSR decreases, with the wedge angle, *α*, and the refractive index, *n*, growing until the last inflection points.

In addition, BSR with larger *h* are higher than those with lower *h*, but the amplification reduces gradually with the growth of *h*. It is worth noting that BSR only grows by 3.7% when *h* grows from 4 to 7. A larger *h* means more sub-pixel samplings of the scene and a larger volume of data than a lower *h* does. Therefore, *h* = 4 seems to be more effective and reasonable than the other values.

## 4. Experiments and Results

### 4.1. Prototype Parameters

**Figure 7:**
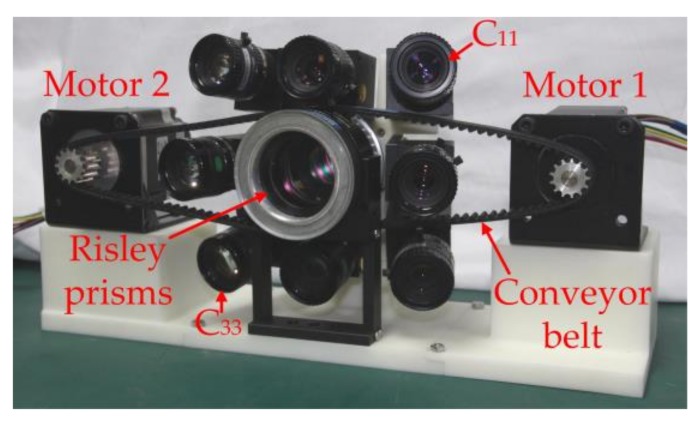
The hybrid bionic image sensor (HBIS) prototype.

A prototype was developed, and the main parameters are shown in Table 1. The FOV of the single ommatidium was 22.6° × 17.1°. Based on the analysis above, the key parameters of HBIS were *φ_h_* ≤ 18.4°, *φ_v_* ≤ 12.9° and 1 ≤ *h* ≤ 7. The prototype employed commercial off-the-shelf cameras, and mechanical assembly and 3D printing techniques were used for the frame, of which errors from size and assembly were inevitable. In order to avoid loss of scene, the parameters were set as *φ_h_* = 15°, *φ_v_* = 10° and *φ_c_* = 17.8°. The prototype is shown in Figure 7. The two prisms were driven by two stepping motors, respectively, and the prisms and stepping motors were connected by conveyor belt. The stepping motors were controlled by a computer through serial ports.

### 4.2. Experimental Results

**Figure 8:**
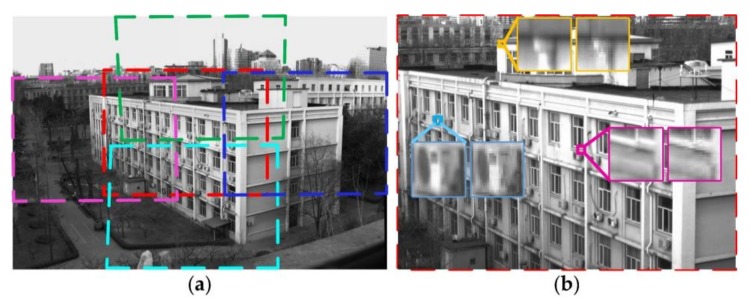
The stitched image with extended field of view (FOV) (**a**) and the super-resolution image of the fovea (**b**). The colored rectangles with dashed lines denote the FOV covered by ommatidia of C_12_, C_21_, C_23_ and C_32_ in (**a**), respectively. For the three pairs of local regions in (**b**), the left ones are pieces of the super-resolution image, and the right ones are pieces of a sub-image.

An outdoor scene was sampled to verify the proposed imaging method, and the resolution improvement factor was set as *h* = 4. Figure 8a shows the stitched image of the prototype. The irregular rectangles of the sub-images mapped by projection transformation were caused by errors in 3D printing and assembly. The whole FOV was 49° × 34°, and the FER*_v_* and FER*_h_* of the prototype were 2.0 and 2.2, respectively, which are verified by Figure 8a. The experimental results for the FOV extension were consistent with the theoretical values: FER*_v_* = 2.2 and FER*_h_* = 2.3. Figure 8b shows the super-resolution image in the fovea. The three pairs of local regions were located in the central and periphery regions of the super-resolution image. We can see that more details of the scene are restored, and less artifacts are retained after the super-resolution reconstruction.

**Figure 9:**
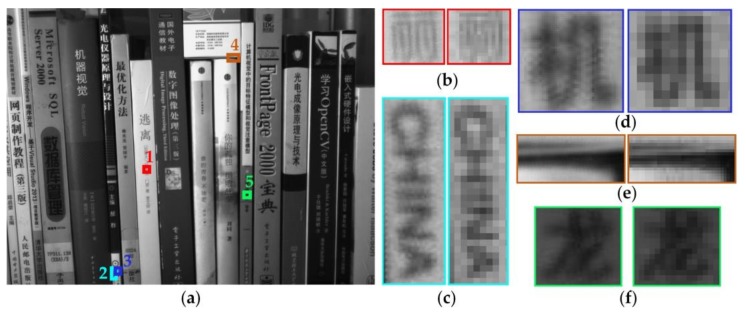
An indoor super-resolution image of the fovea; (**a**) shows the super-resolution image; the left columns of (**b**–**f**) correspond to the local regions of (**a**) marked as 1 to 5, and the right columns of (**b**–**f**) come from the matched regions of one sub-image.

To further explore the scene resolving capability of the fovea, an indoor experiment was carried out with *h* = 4, and the results are shown in Figure 9. From Figure 9b–f, we can see that the quality of the reconstructed image was greatly improved. In Figure 9b–d, the characters in the sub-image are hard to recognize, but they are legible enough to be distinguished after super-resolution reconstruction. Also, the super-resolution image has sharper edges than the sub-image, as shown in Figure 9e. In addition, from Figure 9f, we can see that the super-resolution method performs well in restoring the scene even in low contrast regions.

To summarize, we used a 3 × 3 camera array constituting a HBIS, to achieve FER > 2, an adjustable foveal ratio over a large range (from 1 to 7) and fovea moving capability. Its performances were in accordance with the schematic design and the simulations, including those for FOV extension, super-resolution and foveated imaging. We used less ommatidia to achieve comparable properties than the foveated imaging system with a 5 × 5 camera array [15].

## 5. Discussion

From the analysis above, we can deduce that the capacity of Risley prisms to deviate the FOV of the fovea is key for improving the performance of FOV extension, as shown in Figure 1c and Figure 4. Therefore, it is necessary to discuss the relationship between the deviated FOV of the fovea and FOV extension of the HBIS. For the situation in Figure 1c, the case has been studied preliminarily, in which the bottom margin of FOV of the fovea is below the original optical axis of the fovea. Thus, we further discuss different situations here.

For the situation in Figure 1c, the fovea contributes part of its FOV to the whole FOV of the HBIS. This situation corresponds to the curves before the inflection points in Figure 4. When there are no suspected objects detected, all the ommatidia, including the fovea, work on object detection, and only the original resolution images are sampled by the fovea without super-resolution reconstruction. Once suspected objects are found, the fovea are redirected to the objects by rotating the Risley prisms [33,34], and super-resolution imaging is then achieved.

The other situation shown in Figure 1c is that the lower edge of FOV of the fovea is beyond the initial optical axis of the central camera before the Risley prisms. This situation corresponds to the curves after the inflection points in Figure 4, in which a large wedge angle and refractive index would not change the whole FOV of HBIS. In this situation, the fovea only focuses on high level tasks such as salient object recognition and object tracking without contributing to FOV extension. As shown in Figure 5b,c we can see that larger wedge angle and refractive index values lead to larger image distortion than smaller values do. Therefore, there is no need to adopt larger values for the wedge angle and refractive index than the values at inflection points.

**Figure 10:**
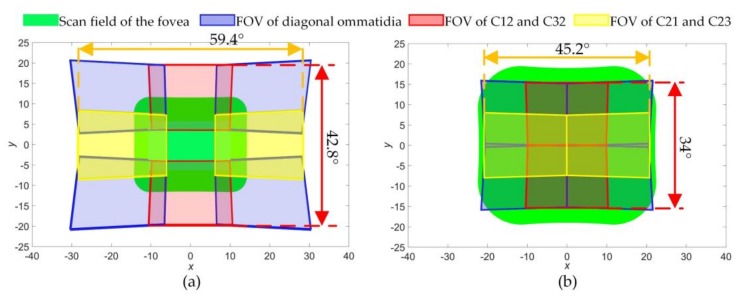
Field of view (FOV) distribution of periphery ommatidia and fovea scan field. (**a**) *φ_h_* = 18.4°, *φ_v_* = 12.9°, *α* = 4°; (**b**) *φ_h_* = 11.3°, *φ_v_* = 8.5°, *α* = 11°.

Taking *α* = 4° and the inflection point *α* = 11° of Figure 4b as two examples of different situations above, the FOV distributions of ommatidia were simulated and shown in Figure 10. *φ_h_* and *φ_v_* were set according to the upper limits (the red line) of Figure 4b. In Figure 10a, the HBIS achieves FER*_v_* = 2.5 and FER*_h_* = 2.6, while the fovea can only scan part of the whole FOV. In Figure 10b, the HBIS achieves FER*_v_* = FER*_h_* = 2 which is smaller than in Figure 10a, but the scan field of the fovea covers the whole FOV of the HBIS. It is verified that the larger the wedge angles are, the smaller the whole FOV is. The same law exists between the refractive index and the whole FOV. In addition, the fovea can move over the entire FOV of the HBIS for Figure 10b, and this makes the HBIS more outstanding than that presented in ref. [15] which uses a 5 × 5 camera array achieving twice the FOV extension and a foveal ratio of 5.9 without the capability of fovea movement.

## 6. Conclusions and Future Work

To achieve large FOV extension and high-resolution imaging simultaneously, we proposed an HBIS that combines features of CACE, PACE and foveated vision. An ommatidium array was arranged on a curved surface to achieve FOV extension of CACE. Risley prisms were used in front of the fovea for super-resolution imaging of PACE. The spatial variant imaging resolution achieved a large foveal ratio and BSR for foveated vision. In addition, the scan capability of the fovea imitates the fovea movement in human eyes. Simulations showed that by using a 3 × 3 camera array, the FOV can be extended by 2.9 times. Meanwhile, a foveal ratio of up to 7 was achieved, and the BSR was beyond 80%. A prototype was developed using commercial off-the shelf-products, and we obtained 2.3 times FOV extension and 4 times resolution improvement of the fovea using the prototype; these values are consistent with the simulations, proving the potential of HBIS to produce a large FOV and foveal ratio with low costs.

Our previous work designed a compound and human hybrid eye with a micro-lens array for 3D imaging [28,35]. Therefore, the proposed HBIS can also be used for 3D imaging with a large FOV and foveal ratio; this will be studied by our team in the future. In addition, the cellular neural/nonlinear network (CNN) paradigm proved to be an effective way of accelerating the image process for real-time applications [36,37]. In the next step, we will improve the HBIS by using CNN to achieve fast super-resolution reconstruction and object detection.

## Figures and Tables

**Table 1:** The main parameters of the prototype.

Parameter Type	Abbreviation	Values
Pixel pitch	*p*	3.75 μm
Rows × columns of pixel array	*M* × *N*	960 × 1280
Focal length	*f*’	12 mm
F-number	*F*	1.4
Wedge angle	*α*	4°
Object distance	*v*	50 mm
Refractive index	*n*	1.5
